# Distribution Patterns and Intraspecific Spatial Associations of Different Size Classes in *Krascheninnikovia ceratoides* (L.) Gueldenst. Populations Within the Tibetan Antelope Breeding Grounds of the West Kunlun Mountains

**DOI:** 10.3390/plants15203222

**Published:** 2026-10-20

**Authors:** Mengyu Chen, Kailing Huang, Fengbing Lai, Xiang Jia, Dingjie Zhao, Yuanxin Jia

**Affiliations:** 1 Beijing Key Laboratory of Precision Forestry, Forestry College, Beijing Forestry University, Beijing 100083, China; cheny1992@bjfu.edu.cn; 2 Key Laboratory of Forest Cultivation and Protection, Ministry of Education, Beijing Forestry University, Beijing 100083, China; 3 College of Geographical Science and Tourism, Xinjiang Normal University, Urumqi 830054, China; huangkailing839@gmail.com; 4 Xinjiang Key Laboratory of Arid Zone Lake Environment and Resources, Urumqi 830054, China; 5 Institute of Geographical Sciences, Henan Academy of Sciences, Zhengzhou 450052, China; 6 Academy of Forest Inventory and Planning, National Forestry and Grassland Administration, Beijing 100714, China

**Keywords:** Western Kunlun Tibetan antelope breeding grounds, *Krascheninnikovia ceratoides*, spatial point pattern, spatial association

## Abstract

This study analyzed the spatial distribution patterns and intraspecific associations of *Krascheninnikovia ceratoides* populations across four typical sample plots at different elevations in the Tibetan antelope breeding grounds of the West Kunlun Mountains, categorizing individuals by measured crown surface area rather than validated chronological age. This descriptive analysis compares spatial point patterns among size classes and sites. By integrating GIS, remote sensing, and UAV imagery with field surveys, we applied the g(r) function of point pattern analysis and three null models to investigate population distribution characteristics and intraspecific associations. Across the four sites, some patterns showed clustering at short distances and fell within the simulated model envelopes at many larger distances, but the plots differed in size and distance range; fits to inhomogeneous Poisson and Thomas cluster models did not identify the processes responsible for those patterns. At short distances, some small-class point patterns were clustered; the patterns of intermediate and large size classes varied by plot and distance. Small- and intermediate-sized classes showed positive association at some short distances and null or negative association at some longer distances within individual plots, but spatial association alone does not establish cooperation or competition. Site and elevation are confounded, so these differences do not establish an elevational trend.

## 1. Introduction

A population refers to an assemblage of conspecific individuals within a specific spatiotemporal context. The spatial distribution pattern of a population describes the arrangement and spatial characteristics of individuals within a community, reflecting the combined effects of the species’ biological traits, intraspecific and interspecific relationships, and environmental resources [1]. Studying population structure and spatial distribution patterns provides critical insights into the organization and dynamics of single or multiple populations within a community, as well as the ecological traits of the species [2]. Population distributions are generally categorized into random, uniform, and aggregated patterns. Random, regular, and clustered patterns describe point arrangements relative to a specified null model; none alone identifies the ecological process that produced them [3]. Intraspecific spatial association here denotes deviations in the relative locations of size classes from a bivariate null model; positive or negative deviations alone do not demonstrate cooperation or competition [4]. Scale significantly influences the interpretation of spatial patterns. Common methods for analyzing spatial distribution patterns include trend surface analysis, two-way local variance, and species adjacency, which focus on single-scale investigations. In contrast, point pattern analysis enables the study of species population characteristics, intra- and interspecific associations, and spatial-scale variation across multiple distances [5]. This approach treats individuals as points in a two-dimensional plane, constructing spatial distribution maps to analyze point patterns [6].

The Tibetan antelope (*Pantholops hodgsonii*) calving grounds in the western Kunlun Mountains are located on the Qinghai–Tibet Plateau and serve as one of China’s critical breeding sites for this species [7]. Schaller [8] observed that approximately 5000 Tibetan antelopes, primarily from the western subpopulation of the Qiangtang region, migrate here to breed annually between mid-May and mid-July. As a Class I protected wildlife species in China, the Tibetan antelope is endemic to the Qinghai–Tibet Plateau and the only migratory ungulate in the Qiangtang region. It primarily inhabits high-altitude plateaus, hills, and mountain valleys at elevations of 3700–5500 m [9]. *Krascheninnikovia ceratoides* (L.) Gueldenst. (Amaranthaceae), a cushion-forming dwarf semi-shrub, constitutes over 50% of the Tibetan antelope’s diet [10], providing essential nutrients for calf development. This species features prostrate woody branches, elongated root systems, and small elliptical to oblong leaves. It typically grows to heights of 10–25 cm, with annual shoots reaching 1.5–5 cm. Renowned for its tolerance to cold, drought, poor soils [11], and salinity [12], *K. ceratoides* is rich in nutrients [13] and predominantly distributed in the Qilian Mountains (Gansu), Qinghai, Xinjiang, Tibet, and eastern Pamir, inhabiting slopes or gravelly plains at 3500–5000 m [14]. Existing studies on the genus *Krascheninnikovia* have focused on phylogenetic evolution [15], paleoenvironmental indicators [16], and genomic traits [17], with frequent mentions in vegetation surveys of the Qinghai–Tibet Plateau [18]. However, systematic analyses of the spatial distribution patterns and intraspecific spatial associations of *K. ceratoides* across different elevations and size classes remain scarce. Describing these patterns can guide future tests of cushion-plant spatial ecology in Tibetan antelope habitats. This study employs null model-based point pattern analysis to explore the spatial distribution and intraspecific associations of *K. ceratoides* populations among four sites at different elevations and among measured size classes. The findings provide a descriptive baseline, not a causal test of elevation, plant age, or habitat conditions.

## 2. Results

### 2.1. Population Structure Characteristics and Size–Class Distribution of Krascheninnikovia ceratoides

The population structural characteristics of *Krascheninnikovia ceratoides* are summarized in Table 1. Mean height was lowest at S4 (Table 1), but an independent elevation effect cannot be inferred from one site per elevation.

### 2.2. Spatial Distribution Patterns of K. ceratoides Populations

**Figure 1:**
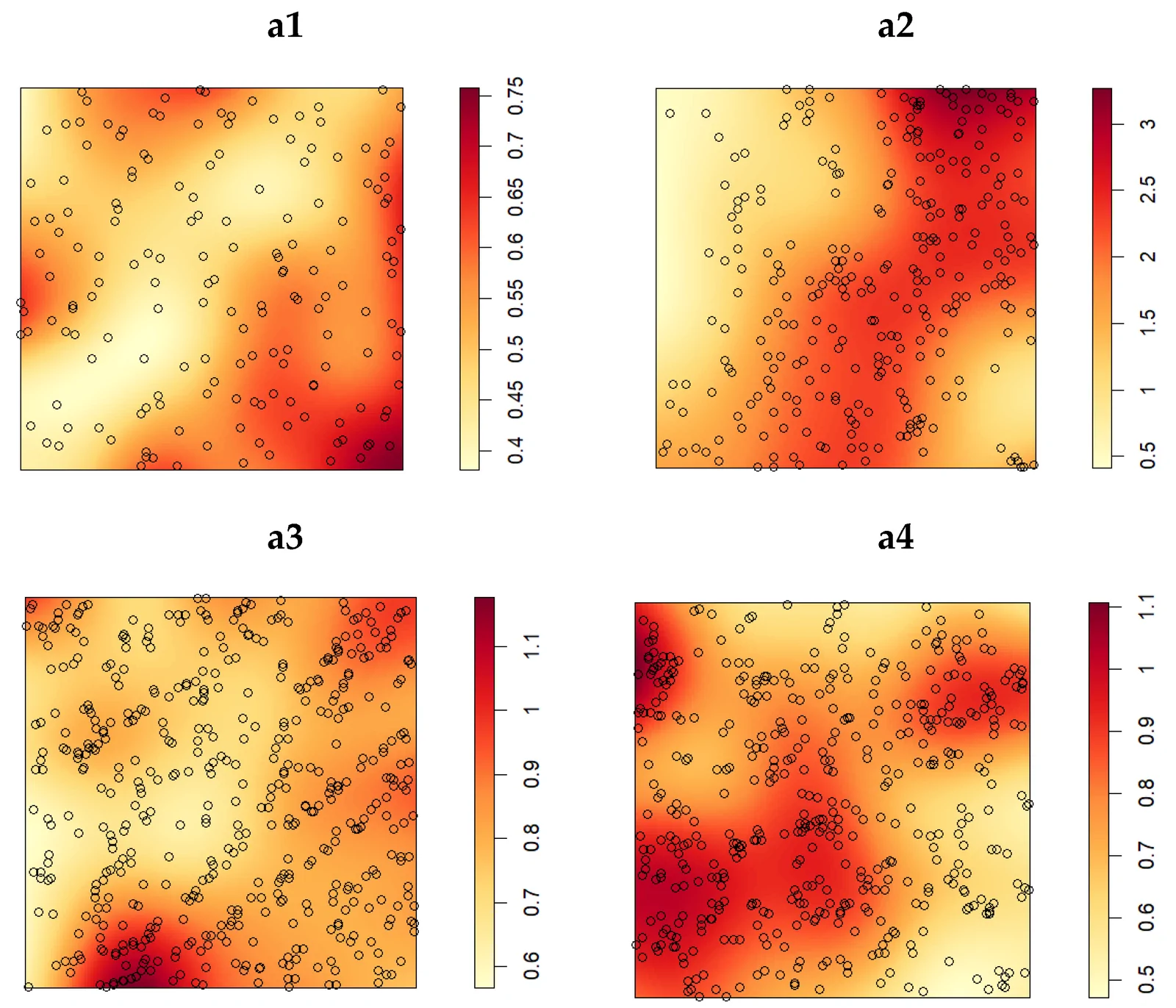
The population density of *K. ceratoides* in various sample plots. Panels (**a1**–**a4**) depict subplots of S1–S4, respectively.

The spatial density distribution of *K. ceratoides* populations is shown in Figure 1(a1–a4). The density of *K. ceratoides* in plot a2 was higher than in other plots at approximately 1.70 plants/m^2^ (288 individuals over 169 m^2^), while Figure 1(a1) contained 171 individuals in 324 m^2^ (approximately 0.53 plants/m^2^), the lowest subplot-wide density. The distribution range varied across plots: *K. ceratoides* in plots a1 and a3 followed a pattern of decreasing density from the periphery toward the center, whereas in plot a2, plants were concentrated in a northeast–south band-shaped range. Plot a4 exhibited denser aggregation in the southwestern area. These differences describe spatial density among four sites at different elevations; they cannot isolate elevation from other site-specific factors.

**Figure 2:**
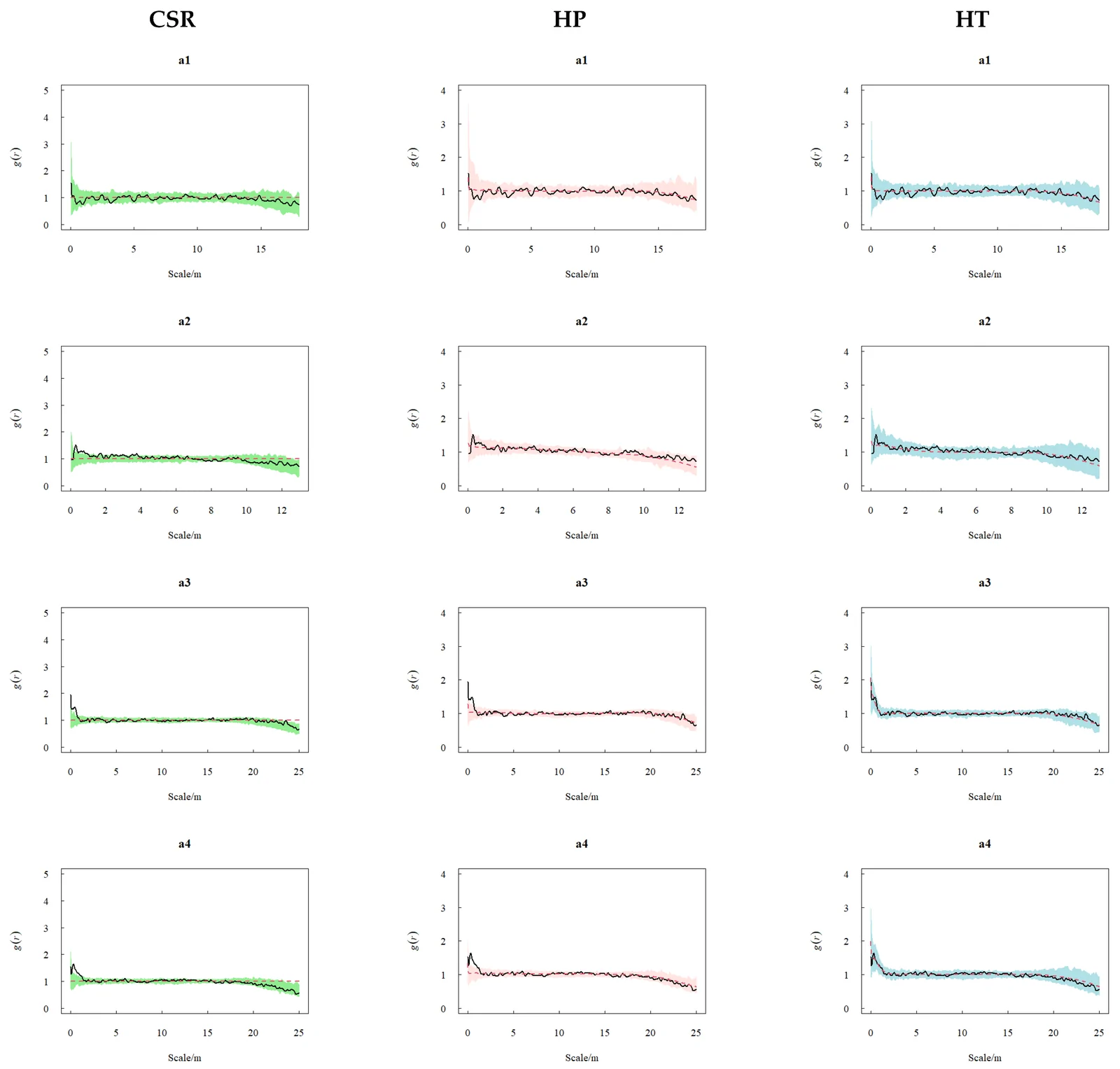
Complete Spatial Randomness (CSR), Inhomogeneous Poisson process (HP), and Homogeneous Thomas process (HT) for *K. ceratoides* populations at different elevations. Panels (**a1**–**a4**) depict subplots of S1–S4, respectively. Black solid curves show observed g(r); red dashed curves show g(r) = 1 (CSR) or simulated means (HP and HT). Shading indicates pointwise simulation envelopes: green (CSR), pink (HP), and light blue (HT).

Point-pattern analysis under CSR, HP, and HT (Figure 2) showed that, relative to CSR, plot-specific departures were concentrated at short distances. The a1 plot fell within the CSR envelope at the reported distances, while a2, a3, and a4 plots showed aggregated distribution at 0.2–1 m, 0.15–1.2 m, and 0.1–1.4 m scales, respectively, and fell within the respective model envelope at other reported distances. Under the inhomogeneous Poisson (HP) null model, plants in a1 and a2 plots fell within its simulated envelope over the reported distances, whereas a3 and a4 plots displayed aggregated distribution at very small scales (a3: 0.2–1.1 m; a4: 0.18–1.5 m) and fell within the respective model envelope at other reported distances, but the cause of spatial intensity variation was not established. Under the homogeneous Thomas process (HT), the observed patterns generally fell within its simulated envelopes at the reported distances, without establishing a specific dispersal mechanism.

### 2.3. Distribution Patterns and Intraspecific Associations of K. ceratoides Across Different Size Classes

#### 2.3.1. Density and Distribution Patterns of *K. ceratoides* Across Different Size Classes

**Figure 3:**
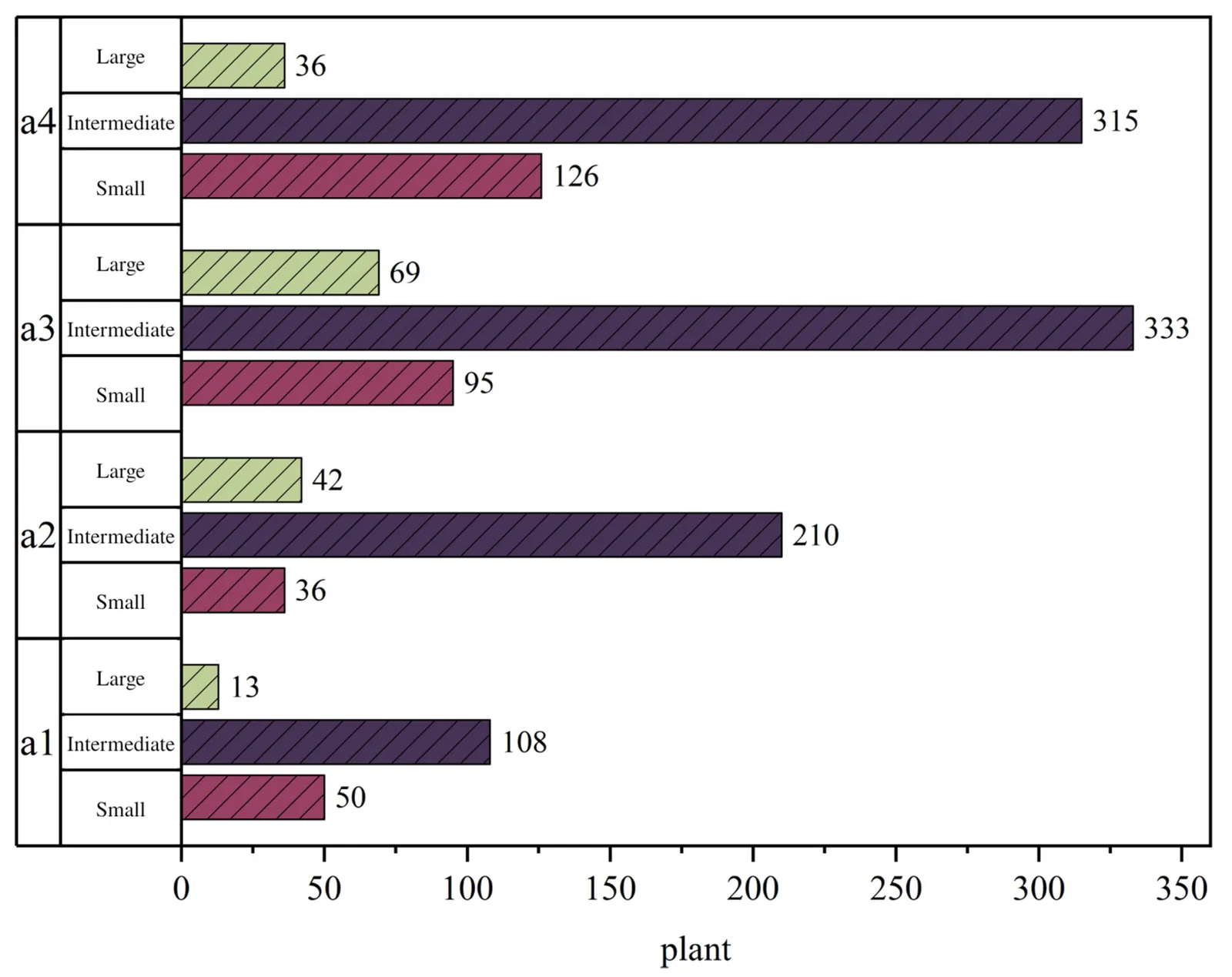
Size–class composition of *K. ceratoides* in various sample plots. Panels (**a1**–**a4**) correspond to subplots within S1–S4, respectively.

**Figure 4:**
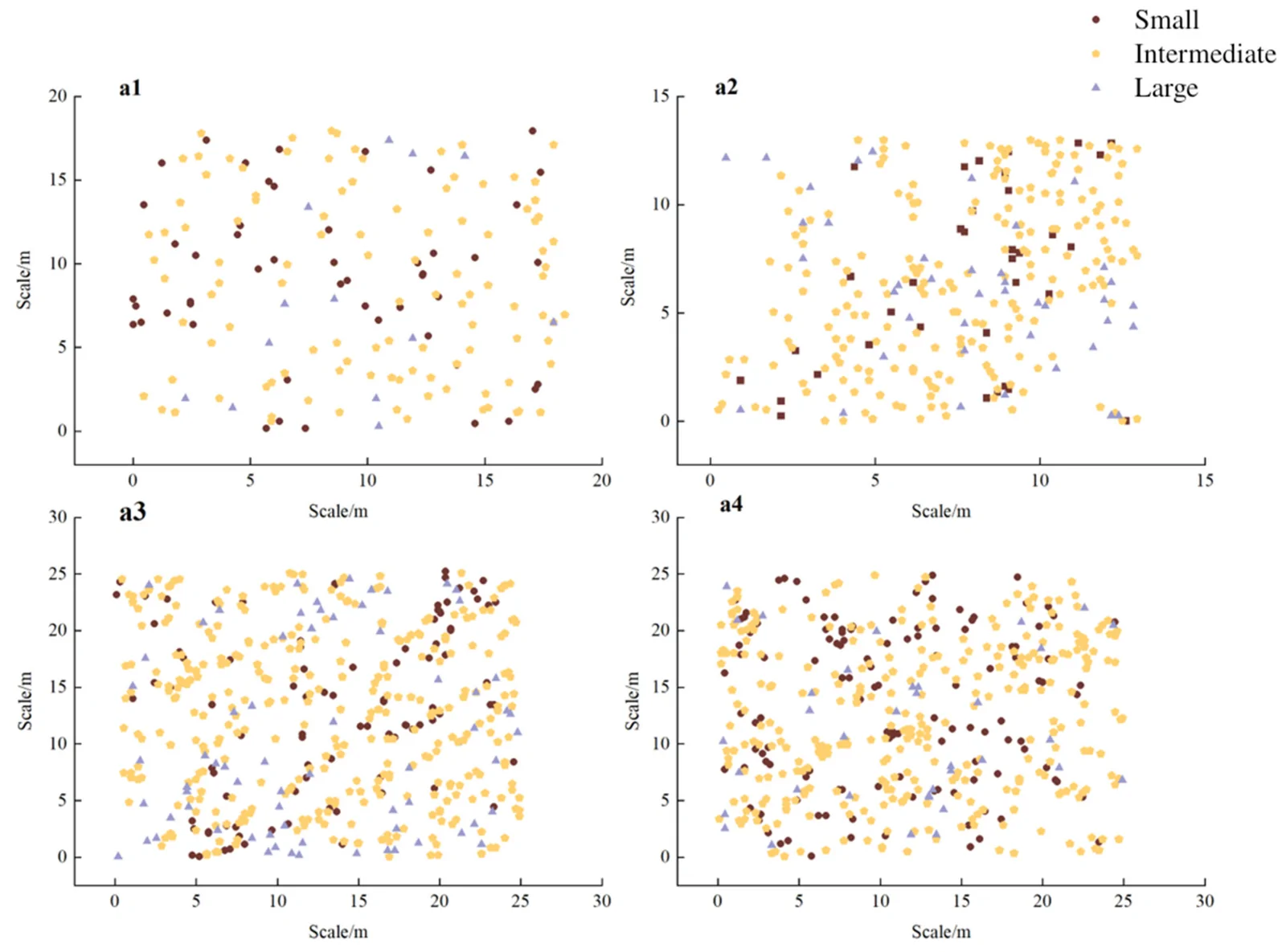
Population distribution patterns of *K. ceratoides* across various sample plots. Panels (**a1**–**a4**) correspond to subplots within S1–S4, respectively. Each marker denotes one individual. Brown circles (a1, a3, and a4) or squares (a2), yellow pentagons, and purple triangles represent the small, intermediate, and large size classes, respectively.

*K. ceratoides* individuals were classified by crown surface area into size classes (results shown in Figure 3). Plot a1 contained 50 small, 108 intermediate-sized, and 13 large individuals; plot a2 had 36 small, 210 intermediate-sized, and 42 large; plot a3 included 95 small, 333 intermediate-sized, and 69 large; and plot a4 comprised 126 small, 315 intermediate-sized, and 36 large. Intermediate-sized individuals dominated all plots, accounting for 63.16%, 72.92%, 67%, and 66.04% in a1–a4, respectively. Large individuals exhibited the lowest proportions in all plots except a2, where they slightly outnumbered small individuals. The large-class proportions were 7.60% in a1, 14.58% in a2, 13.88% in a3, and 7.55% in a4. Pooled across plots, the intermediate class was most numerous, followed by the small and large classes; this single size-based census does not establish population stability. In Figure 4, population distribution patterns showed that individuals in plot a1 were relatively scattered across size classes, while those in a2–a4 were more clustered. Small individuals primarily overlapped with intermediate-sized distribution areas, whereas large individuals were more dispersed.

Counts and mapped locations of size classes are shown in Figure 3 and Figure 4, respectively. Intermediate-sized individuals were more numerous than the other size classes in each plot. In a2, the intermediate class was especially numerous (210 individuals); a1 contained 108. Intermediate-sized plants in plots a1 and a3 were primarily clustered along the periphery, whereas those in plots a2 and a4 were concentrated in north–south strip-shaped zones and the eastern–western regions, respectively, with plot-specific concentration patterns. Small-class counts were lower than intermediate-class counts in every plot. In plot a1, small individuals occurred in local patches; in plots a2 and a3, they were concentrated in the northeastern and southern areas. Plot a4 displayed a decline in the local concentration of small individuals from northwest to southeast, with higher concentrations in the western zone. Large-class counts were lowest in three of the four plots. In plots a1 and a3, large individuals clustered in northern and southern ranges, while in plot a2, they occurred in central and eastern areas, and in plot a4, they occupied central and southern zones. Pooled counts followed the order intermediate > small > large, while mapped locations varied among the four sites; an independent elevation effect could not be estimated.

**Figure 5:**
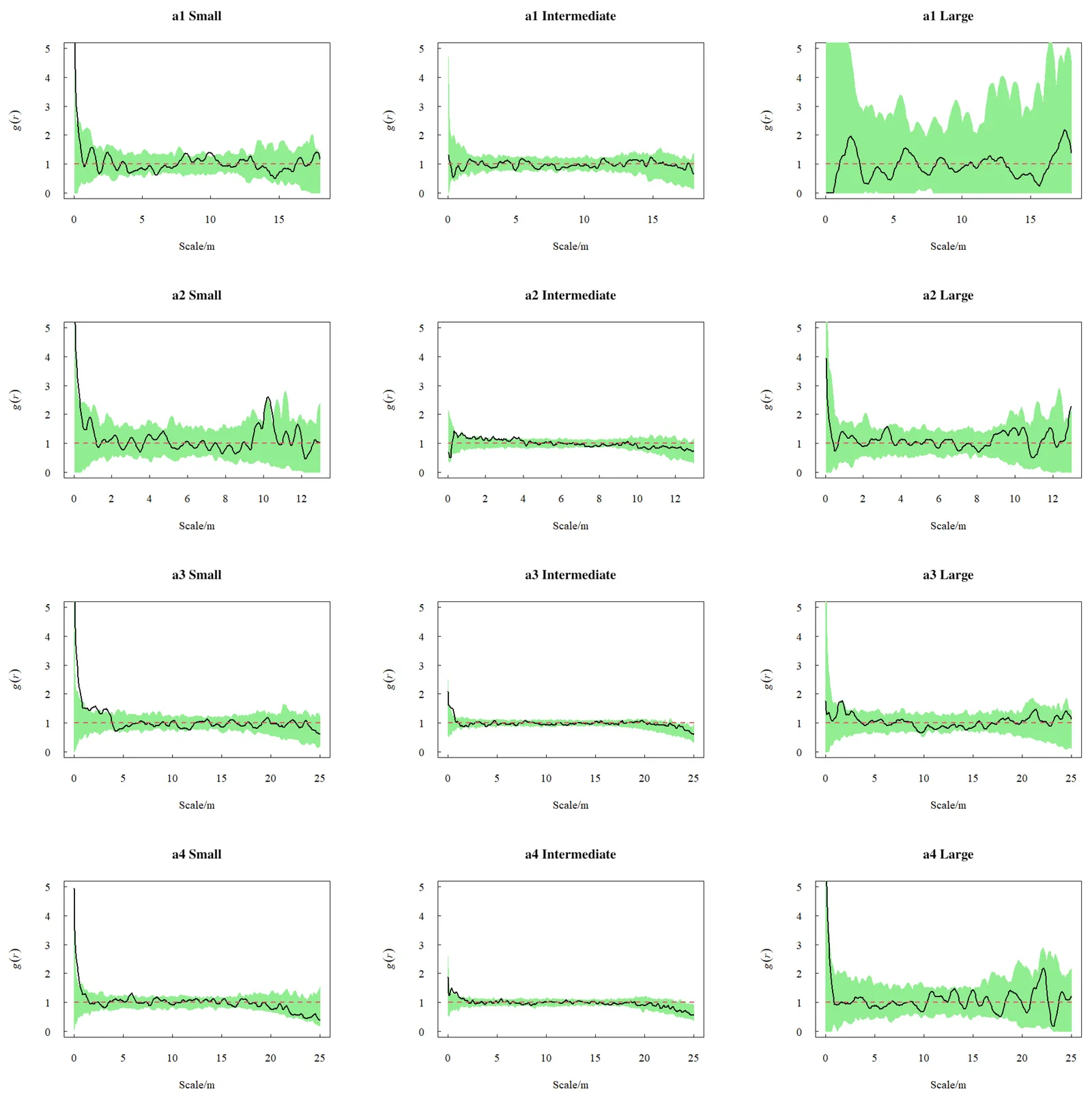
Spatial distribution patterns of *K. ceratoides* across size classes in sample plots based on CSR. Panels (**a1**–**a4**) correspond to subplots within S1–S4, respectively. Black solid curves show the observed g(r), red dashed lines show the CSR reference g(r) = 1, and green shaded areas show the pointwise simulation envelopes under CSR.

**Figure 6:**
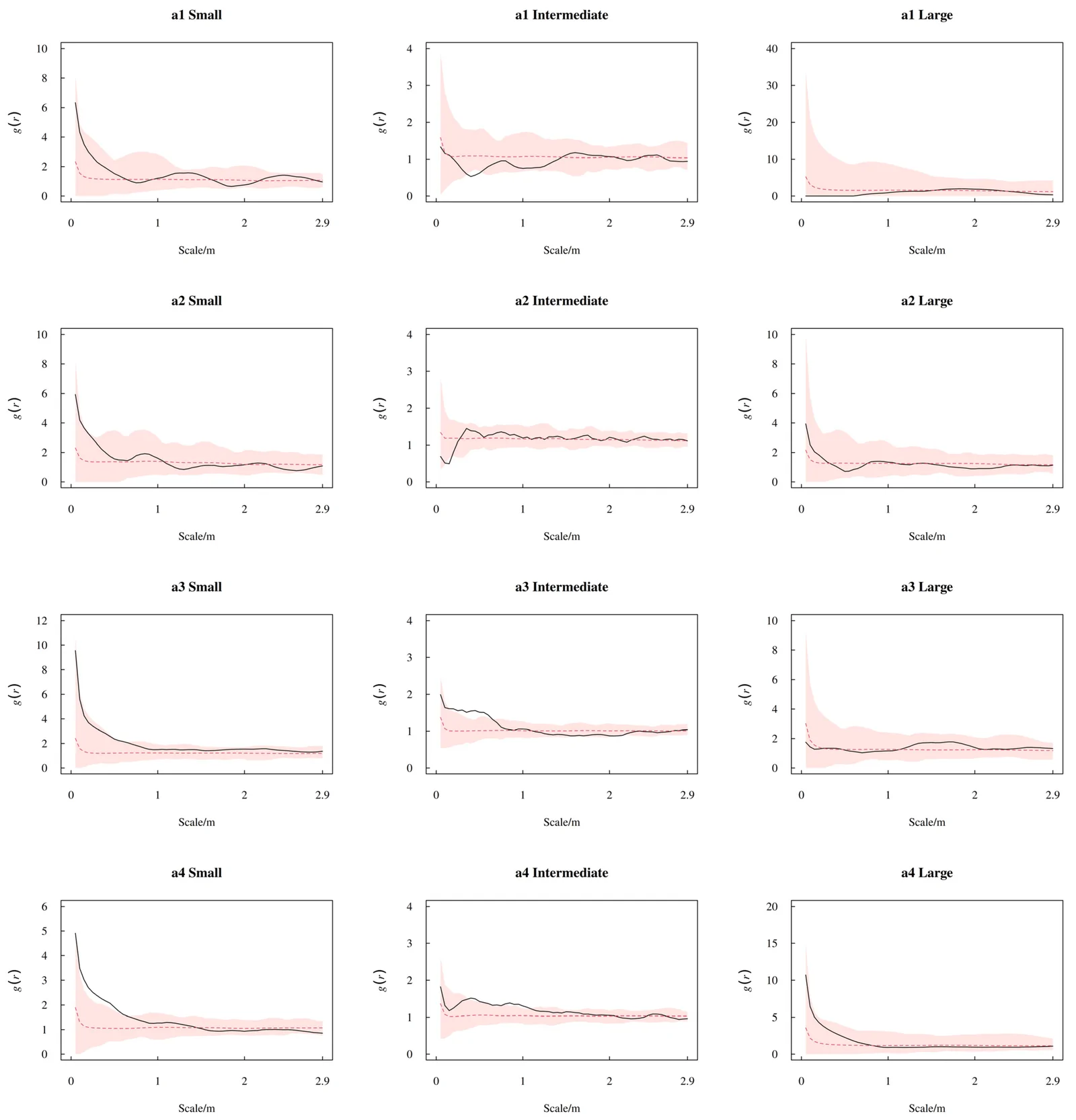
Spatial distribution patterns of *K. ceratoides* across size classes in sample plots based on HP. Panels (**a1**–**a4**) correspond to subplots within S1–S4, respectively. Black solid curves show the observed g(r), red dashed curves show the mean g(r) from 199 simulations of the fitted inhomogeneous Poisson model, and pink shaded areas show the pointwise minimum–maximum simulation envelopes. Comparisons are restricted to 0.05–2.9 m.

**Figure 7:**
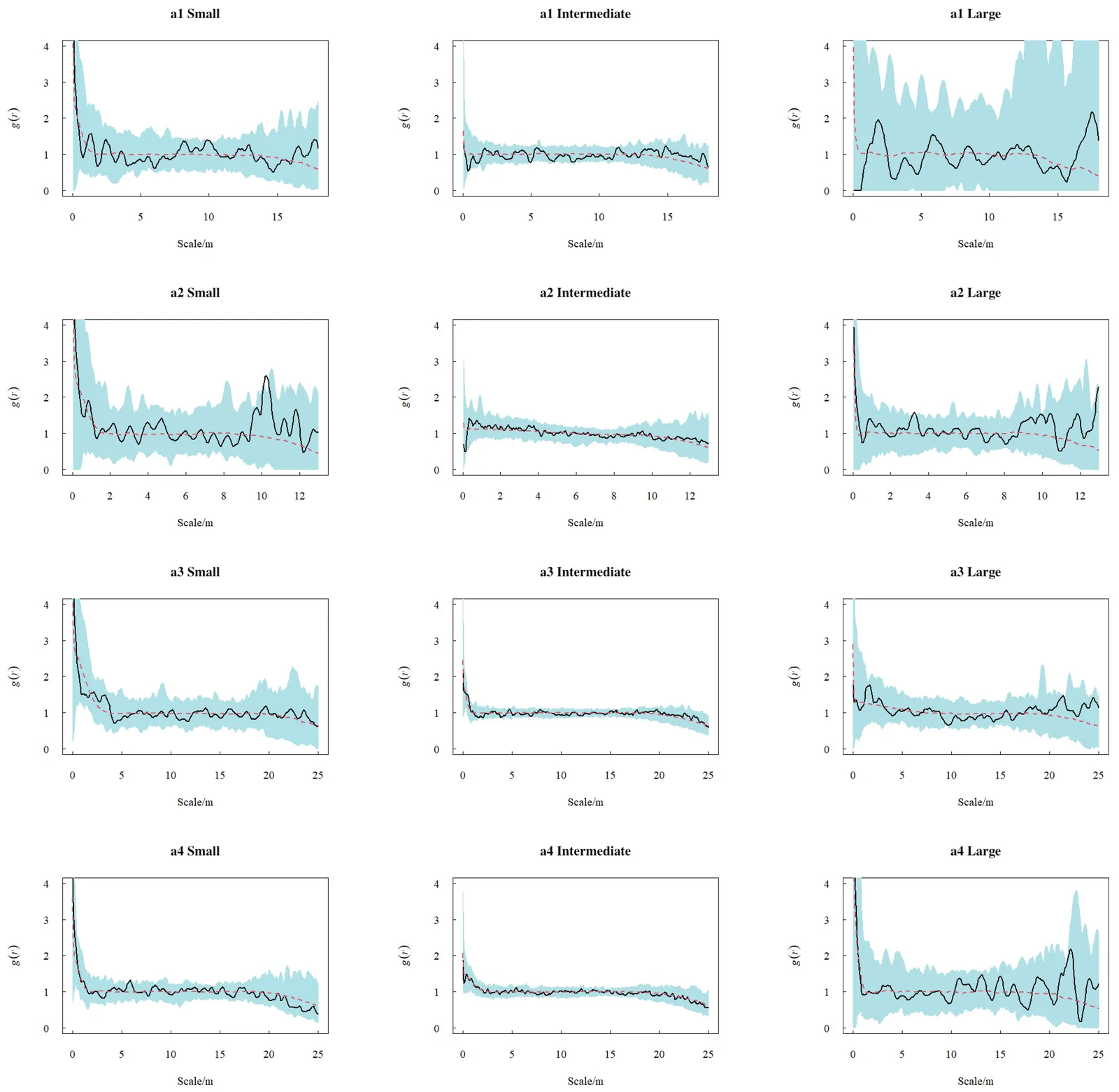
Spatial distribution patterns of *K. ceratoides* across size classes in sample plots based on HT. Panels (**a1**–**a4**) correspond to subplots within S1–S4, respectively. Black solid curves show the observed g(r), red dashed curves show the mean simulated g(r) under the fitted homogeneous Thomas model, and light-blue shaded areas show the pointwise simulation envelopes.

The spatial distribution analysis of *K. ceratoides* across different size classes and sample plots yielded the following results: Under the CSR (Figure 5), small plants exhibited clustered distribution at small scales in all plots. Plots a1 and a2 displayed similar distribution patterns, showing clustering at both small and intermediate scales (a1: 0.1–0.18 m, 8.3–8.8 m, and 9.2–10 m; a2: 0.1–0.15 m and 9.5–10.5 m). Plot a3 was predominantly clustered at positive distances up to 4.1 m, while plot a4 showed clustering at 0.05–0.11 m. Intermediate-sized patterns in plots a1 and a2 fell within CSR envelopes at reported distances, whereas plots a3 and a4 displayed clustered distribution at very small scales (a3: 0.12–0.16 m; a4: 0.11–0.8 m). Large-class patterns generally fell within CSR envelopes at the reported distances. In the recomputed size–class HP analysis, comparisons were restricted to the common distance grid of 0.05–2.9 m (Figure 6). The observed g(r) of large plants at all four sites remained within the pointwise HP simulation envelopes. Small and intermediate plants at a3 and a4 exceeded the upper envelope at selected short distances; intermediate plants at a1, a2, and a3 fell below the lower envelope at selected distances. These departures describe differences from a Poisson model conditional on fitted spatially varying intensity, not environmental causes or plant interactions; remaining within an envelope does not establish spatial randomness. Under HT (Figure 7), class-specific patterns generally fell within the simulated envelopes at the distances shown; this does not establish a dispersal mechanism.

#### 2.3.2. Observed Spatial Associations Between Size Classes

**Figure 8:**
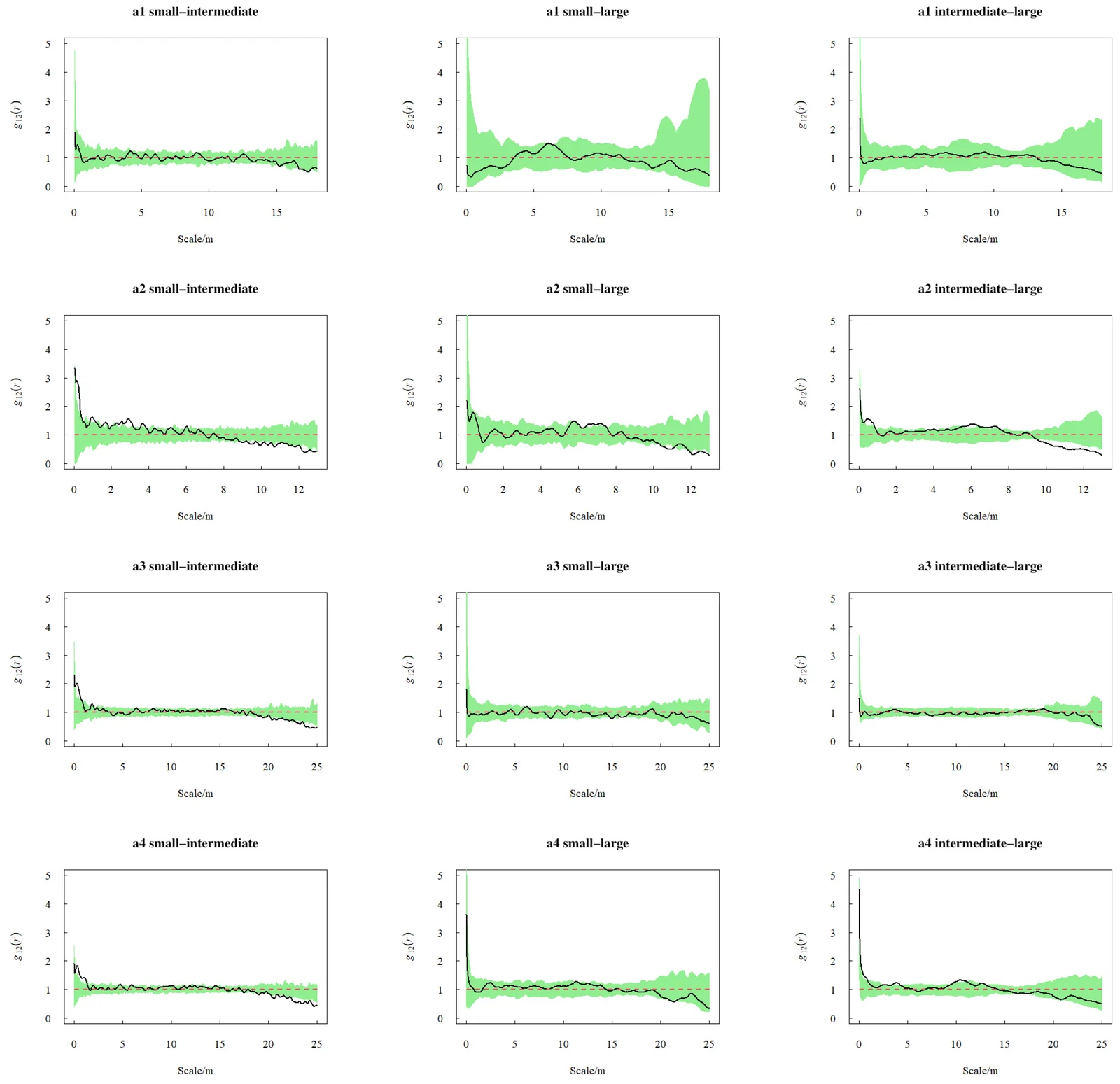
Intraspecific association of *K. ceratoides* across size classes in sample plots under bivariate CSR. Panels (**a1**–**a4**) correspond to subplots within S1–S4, respectively. Black solid curves show the observed cross-type pair-correlation function $g_{12}(r)$, red dashed lines show the theoretical reference $g_{12}(r)$ = 1, and green shaded areas show the pointwise minimum–maximum envelopes from 99 simulations of two independent homogeneous Poisson processes.

As shown in Figure 8, the intraspecific association between small- and intermediate-sized plants varied across the spatial distances examined. In plot a1, the function remained within the simulated envelope at the reported distances. Plots a2, a3, and a4 exhibited transitions from positive to neutral and finally negative associations: a2 showed predominantly positive association at scales <3 m but negative association at >8.5 m; a3 displayed positive association at 0.1–1.5 m and negative association at 20–21.8 m and 22.4–25 m; a4 manifested positive association at 0.6–2 m and negative association at 20.5–25 m. For small–large associations, plot a1 exhibited positive association at intermediate scales (6–7 m); plot a2 showed positive association at 6.5–7.5 m but shifted to predominantly negative association at >10.5 m; the functions for plots a3 and a4 stayed within the simulated envelopes at the reported distances. Regarding intermediate-sized–large associations, the functions for plots a1 and a3 remained within the simulated envelopes at the reported distances; plot a2 demonstrated positive association at small to intermediate scales but distinct negative association at >9.5 m; plot a4 exhibited alternating positive departures and values within the envelope at scales <10 m, and then remained within the envelope beyond this threshold. Some pairs showed positive association at short distances and null or negative association at longer distances within individual plots. Because observation windows differ, long-distance patterns should not be compared directly among sites.

## 3. Discussion

### 3.1. Population Characteristics and Spatial Distribution Patterns of K. ceratoides

Along elevational gradients, various environmental factors including precipitation, solar radiation, temperature, and wind speed change significantly over short distances, directly or indirectly affecting plant diversity and community composition. On the Qinghai–Tibet Plateau specifically, conditions feature large diurnal temperature variation, low precipitation, low temperatures, high wind speeds, prolonged snow cover periods, and soils deficient in organic matter and nitrogen with predominantly alkaline pH [19]. Under these extreme conditions, plants have evolved diverse ecological traits through long-term adaptation, developing homeostatic mechanisms and unique adaptive traits. Size–class composition and spatial point patterns describe this census, but do not establish recruitment, mortality, population dynamics, or community succession. Spatial distribution patterns reflect biological characteristics, intra- and interspecific competition, disturbances, and habitat heterogeneity, representing the horizontal-scale distribution of individual plants [20]. This study established sample plots across elevational gradients in the Western Kunlun Mountains to analyze population characteristics and spatial distribution patterns of *K. ceratoides* in Tibetan antelope breeding habitats. Intermediate-sized and small individuals were more common than large ones in these plots; a single size-based census does not establish population stability. Size–class point patterns varied by plot and spatial distance. Warming may alter alpine vegetation [21], but temporal vegetation change and replacement of *K. ceratoides* were not examined in this study.

Under CSR, plot a1 showed no reported departure and a2–a4 showed clustering at certain distances; these site-specific observations do not establish an elevation effect. This aligns with Zhao et al.’s findings [22] on *Thylacospermum caespitosum* (Cambess.) Schischk distribution patterns in the Kunlun Pass, although resource requirements were not measured here. Clonal growth may generate clustering in some cushion plants [23], but clonal recruitment was not measured here. Clustering alone does not demonstrate increased survival or stress resistance. We did not measure competition, resource availability, or self-thinning; scale-dependent fit should not be interpreted as evidence for those processes [24,25]. The lack of detectable uniformity does not identify its ecological cause [26,27]. Observed scale dependence does not by itself establish effects on community diversity [28]. In the recomputed size–class HP analyses, large-class curves remained within the pointwise simulation envelopes over 0.05–2.9 m; these comparisons are conditional on spatial intensity fitted from the observed plants and do not identify habitat effects. At a3 and a4, small and intermediate plants exceeded the upper HP envelope at selected short distances; intermediate plants at a1, a2, and a3 fell below the lower envelope at selected distances. Under HT, the patterns generally fell within the simulated envelopes at the reported distances. A fit to a cluster process does not by itself establish reproductive dispersal. The other plots generally fell within the HT envelopes as well. These results cannot partition effects of site, elevation, habitat, and dispersal on the observed point patterns. Plot-level soil moisture, temperature, and nutrients were not measured; each elevation had one site, and observation windows differed in area and distance coverage. Mechanistic hypotheses require independent measurements and replicated analyses.

### 3.2. Spatial Distribution Patterns of K. ceratoides Across Size Classes

Spatial patterns differed among measured size classes [29], but size does not establish plant age or the processes generating those patterns. This study analyzed small, intermediate-sized, and large plants using CSR, HP, and HT. Under CSR, short-distance clustering was reported more often for small than large plants in these plots; the classes do not represent observed transitions through time. Related point-pattern studies provide context for these contrasts [30].

Small plants showed clustering at some short distances, but survival and stress tolerance were not measured [31]. Class frequencies from a single census cannot distinguish recruitment, growth, or mortality [25]. Several class-specific patterns fell within the fitted HP envelopes over 0.05–2.9 m or within HT envelopes at their originally reported distances; neither comparison identifies habitat features or reproduction. Hydrothermal conditions and clonal propagation were not measured, so patterns in a3 cannot be assigned to these mechanisms. The available data do not establish size changes or diffusion processes.

### 3.3. Intraspecific Association of K. ceratoides Across Size Classes

For some size–class pairs, association changed with distance within plots; differences between sites cannot be attributed to elevation because each elevation has one site and observation windows differ. Long-distance associations reported for a3 and a4 cannot be directly compared with a1 and a2, whose smaller observation windows do not support those distances. Positive spatial association is not direct evidence of cooperation under environmental stress. Potential nurse-plant effects require independent microhabitat and survival measurements. The study did not measure changes in stress tolerance or resource demand with size, and spatial associations do not establish increased competition [27]. Small–large pairs were often indistinguishable from the reference model at the reported distances; size-dependent interactions were not measured [32]. Clonal transitions between size classes, ecological niches, and resource use were not measured, so null model deviations do not demonstrate mutual non-interference [33]. Agreement with a reference model does not establish the absence of interaction. In a2, several pairs had positive departures at shorter distances and negative departures at longer distances within the plot’s available range. Plot a2 had a high reported density (4548 m elevation), but plot-level environmental drivers were not measured. Local density and fitted intensity may influence the pattern; competition cannot be inferred without independent measurements.

## 4. Materials and Methods

### 4.1. Study Area Description

**Figure 9:**
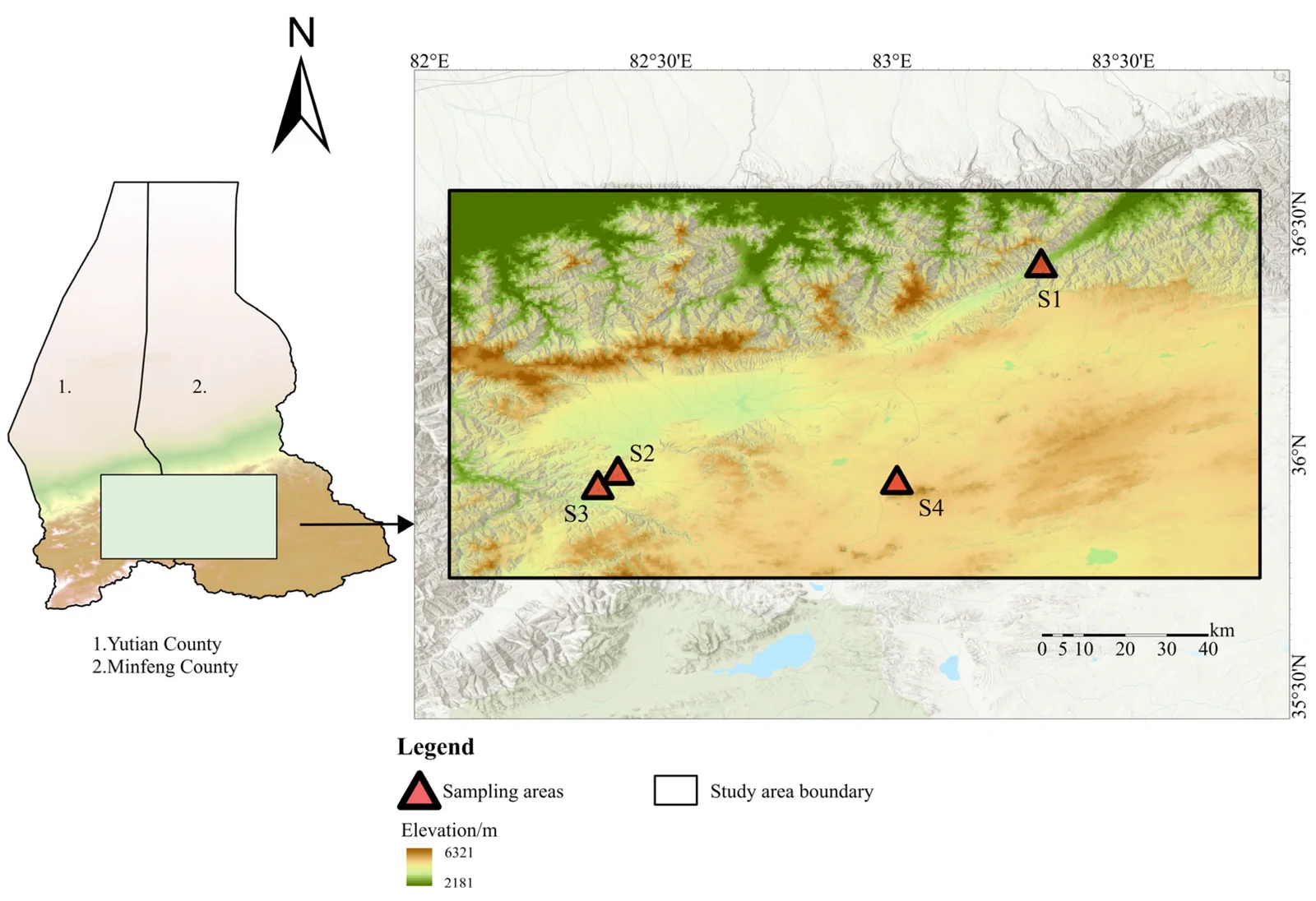
Geographical map of the research area.

The study area is located in the Tibetan antelope breeding grounds of the Western Kunlun Mountains, with an average elevation exceeding 4600 m. Situated on the Qiangtang Plateau, it borders the Ngari Prefecture of Tibet to the south, Minfeng County of Xinjiang to the east, and Yutian County of Xinjiang to the west, spanning approximately 82°2′34.447″–83°47′43.544″ E and 35°44′1.572″–36°34′14.023″ N. This region experiences scant precipitation and extreme cold, forming an alpine desert climate. The warmest month records only 14–20 mm of rainfall, with annual precipitation ranging from 54–95 mm. Average temperatures range from −21 to −17 °C in the coldest month and 5–9 °C in the warmest month, with an annual mean of −18–11 °C (study area overview shown in Figure 9). Most vegetation exhibits a cushion-like morphology—low-growing, densely branched, and with tightly packed leaves—an evolutionary adaptation to extreme conditions including frigidity, intense solar radiation, drastic diurnal temperature shifts, and strong winds. Cushion plants dominate as the predominant ecotype in this alpine desert. Field surveys confirm *K. ceratoides* as the dominant species, forming relatively simple communities with sparse companion species such as *Androsace umbellata* (Lour.) Merr and *Oxytropis microphylla* (Pall.) DC. Soils are primarily alpine desert soil, Aridisols, and calcic soils, with partial saline–alkali patches [14]. Four study plots were established on sunny slopes at representative *Krascheninnikovia ceratoides* habitats along an elevational gradient (4184 m, 4548 m, 4855 m, and 5124 m). Combining drone remote sensing with ground surveys, this research quantifies the distribution characteristics of *K. ceratoides* across the study area.

### 4.2. Data Processing

#### 4.2.1. Sample Plot Establishment

Based on pre-survey route planning, field investigations were conducted from July 29 to 30, 2023. Four monitoring plots (designated S1–S4) were established at elevations of 4184 m, 4548 m, 4855 m, and 5124 m within minimally disturbed *K. ceratoides* habitats (Table 2). Due to challenges in establishing large plots while ensuring image overlap ratio and interpretation accuracy, plot dimensions were adjusted according to environmental heterogeneity, vegetation distribution, topography, and density to capture key ecological characteristics: S1 and S2 measured 30 × 30 m; S3 was 70 × 70 m; and S4 spanned 100 × 100 m.

#### 4.2.2. Point Pattern Data Measurement and Processing

**Figure 10:**
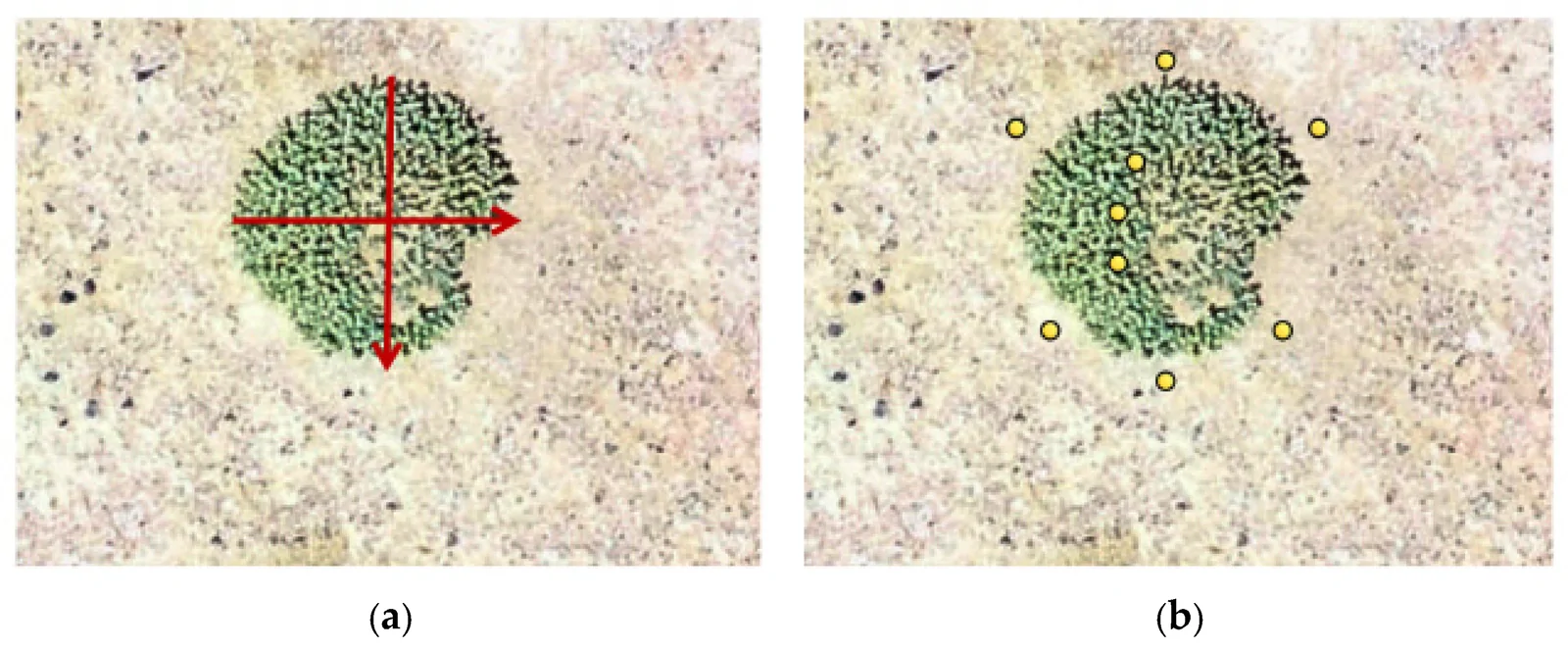
Morphological measurements derived from DOM and DSM: (**a**) crown diameter; (**b**) plant height. Red arrows in (**a**) indicate the two perpendicular crown diameters. Yellow circles in (**b**) indicate elevation-sampling locations within the crown and on the surrounding bare ground used to calculate plant height.

Aerial imagery was captured using a DJI Phantom 4 Pro drone (SZ DJI Technology Co., Ltd., Shenzhen, China) (flight altitude: 150 m; shooting interval: 5 s). Digital Orthophoto Maps (DOMs) and Digital Surface Models (DSMs) were generated through Agisoft Metashape photogrammetry (Agisoft LLC, St. Petersburg, Russia). The orthophoto ground sampling distance was 2.2 cm per pixel; spatial processing used the WGS 1984 World Mercator projected coordinate system. Given the dwarf stature of *Krascheninnikovia ceratoides*, plant height and crown width measurements were conducted in ArcGIS 10.8 (Esri, Redlands, CA, USA) by integrating DOM and DSM data. Plant height was defined as the vertical distance from ground surface to apical bud, calculated by averaging three repeated elevation measurements at the crown center minus the mean elevation of six surrounding bare ground points. Crown diameter was determined by averaging three repeated measurements along east–west and north–south axes, followed by secondary averaging of these directional means (Figure 10). For point-pattern analysis, subplots (a1–a4, corresponding to S1–S4, respectively) were delineated within the central parts of the original plots. Vector boundaries were available for these subplots and defined their nominal analysis windows. Subplot dimensions were: a1 (18 × 18 m), a2 (13 × 13 m), a3 and a4 (25 × 25 m each). According to field personnel, these central windows were chosen pragmatically at approximately half the corresponding orthophoto length, informed by a review of the related literature; this selection was not a statistical edge correction. No window-size sensitivity analysis was conducted. Using the adjacent lattice method, each subplot was subdivided into 1 × 1 m grids. Grid counts and surveyed plants were: a1 (324 grids, 171 plants), a2 (169 grids, 288 plants), a3 (625 grids, 497 plants), and a4 (625 grids, 477 plants). Individual *K. ceratoides* were identified through visual interpretation. The local (0, 0) origin was the lower-left corner of the first grid and the nominal analysis window, whose sides were parallel to the x and y axes. Spreadsheet x and y values were distances in metres from the left and lower boundaries, respectively, for generating 2D point patterns.

### 4.3. Research Methods

#### 4.3.1. Size–Class Identification of *K. ceratoides*

Crown surface area was used as a descriptive measure of plant size. We calculated crown surface area as shown in Equation (1).

(1) $$S=\pi \frac{a}{2}\cdot \frac{b}{2}$$

where *S* represents the surface area (cm^2^) of *K. ceratoides*, *a* denotes the major axis length of the crown spread, and *b* denotes the minor axis length of the crown spread.

For *K. ceratoides*, using crown surface area and size thresholds adapted from another cushion-plant study [34], we defined three descriptive size classes. The mean crown surface area across plots ranged from 187.52 to 220.97 cm^2^. The classes were small (*S* ≤ 120 cm^2^), intermediate (120 < *S* ≤ 340 cm^2^), and large (*S* > 340 cm^2^); microhabitat, grazing, damage, and individual growth rates may affect crown area, and these thresholds have not been validated as indicators of chronological age or reproductive status in this species.

#### 4.3.2. Point Pattern Analysis

Point pattern analysis is typically influenced by two types of effects: first-order variation in spatial intensity and second-order dependence between points; either can have multiple ecological causes [3]. The univariate pair correlation function g(r), derived from Ripley’s K-function, replaces circular areas with ring-shaped annuli for calculation. This approach counteracts the cumulative influence of small-scale spatial patterns on larger scales observed in Ripley’s K-function as spatial extent increases [4]. Therefore, this study employs the pair correlation function g(r) to analyze the point patterns of *K. ceratoides* within four sites located at different elevations, as formalized in Equation (2):

(2) $$g(r)={\left(2\pi r\right)}^{-1}\frac{dK(r)}{dr}$$

where dK(r) is the differential of the function K(r); dr is the differential of the radius *r*.

Observed pair-correlation functions were compared with envelopes generated from Monte Carlo simulations. Values above a model envelope indicate an excess of pairs at that distance relative to the specified model; values within an envelope do not prove that the generating process is random; values below the envelope indicate fewer pairs than predicted by that model. Interpretation of departures depends on the specified null model and observation window [35]. We used three point-process null models [36]. Under complete spatial randomness (CSR), points occur independently at constant intensity within the observation window [37]. An inhomogeneous Poisson process (HP) allows spatially varying intensity; no environmental variables were measured, so such variation cannot be attributed to measured habitat heterogeneity [38]. The homogeneous Thomas cluster process (HT) is a cluster-generating null model; agreement with it is not proof of reproductive spread or dispersal limitation [39]. The original point-pattern analyses were performed in R 4.4.1. The 12 size–class HP analyses for Figure 6 were subsequently recomputed in R 4.3.3 using the settings detailed below; those settings should not be assumed to apply to the CSR, HT, or whole-population HP analyses.

The size–class HP recomputation estimated a Gaussian-kernel intensity surface separately from the observed plant locations for each of the four sites and three size classes (12 patterns) in plants per square metre of projected coordinate area. For each pattern, the Gaussian intensity bandwidth *σ* was set to one-eighth of the shorter side of its computational window (Table 3). Kernel-mass boundary normalization was used (edge = TRUE, diggle = FALSE), and each intensity image was evaluated on a 128 × 128 grid. The fitted intensity was held fixed when generating 199 inhomogeneous Poisson simulations per pattern (2388 simulations in total); simulated point counts were allowed to vary, with expected count equal to the integral of the fitted intensity over the window. No environmental covariates were measured or incorporated.

The ordinary pair-correlation function g(r) of each observed and simulated pattern used Ripley’s isotropic edge correction, an Epanechnikov kernel, and Stoyan’s bandwidth rule (stoyan = 0.15, bw = NULL, divisor = “r”). The pair-correlation bandwidth is distinct from the Gaussian intensity bandwidth. Pointwise minimum–maximum simulation envelopes were constructed (nrank = 1, global = FALSE). Comparisons used a common distance grid from 0.05 to 2.9 m in 0.05 m increments, below the smallest default maximum radius among the 12 observed patterns (2.922 m); r = 0 was excluded from interpretation. These envelopes are exploratory comparisons conditional on intensity fitted from the same observed points, not 95% confidence bands or evidence of an environmental cause. Calculations used R 4.3.3, spatstat.geom 3.2-8, spatstat.explore 3.2-5, and spatstat.random 3.2-2.

Bivariate pair-correlation function analysis was conducted on *K. ceratoides* across different size classes to describe spatial associations between individuals assigned to different size classes [40]. As shown in Equation (3):

(3) $$g_{12}(r)={\left(2\pi r\right)}^{-1}\frac{dK_{12}(r)}{dr}$$

where 1 and 2 represent different size classes of the *K. ceratoides* population, respectively. When $g_{12}(r)$ lies within the simulated envelope, no departure from the specified bivariate null model is detected at that distance; values above it indicate more cross-class pairs than that model predicts; values below it indicate fewer cross-class pairs. These deviations do not by themselves establish facilitation or competition.

Bivariate spatial patterns were evaluated separately for the small–intermediate, small–large, and intermediate–large size–class pairs at each site. For each pair, the two input patterns were encoded as marks A and B and superimposed within a common rectangular window *W*. The window extended 1 m beyond the combined minimum and maximum X and Y coordinates of the two patterns on each side. Let $n_{A}$ and $n_{B}$ denote the observed counts of the two classes and |*W*| the area of this computational window.

The null model comprised two independent homogeneous Poisson processes with constant intensities $\lambda_{A}$ = $n_{A}$/|*W*| and $\lambda_{B}$ = $n_{B}$/|*W*|. Operationally, the default simulator first generated a homogeneous Poisson pattern with pooled intensity ($n_{A}$ + $n_{B}$)/|*W*| in *W*, and then assigned each generated point an independent A or B mark with probabilities $n_{A}$/($n_{A}$ + $n_{B}$) and $n_{B}$/($n_{A}$ + $n_{B}$), respectively. These fitted intensities and mark probabilities remained fixed across simulations. All point locations were generated anew; neither the total point count nor the count of either class was fixed. The procedure therefore did not preserve the observed locations, within-class clustering, or spatially varying intensity, and did not perform random relabelling of the observed points or random shifts in one class.

The original envelope call used 99 simulations through the default nsim setting, with simulate = NULL, fix.n = FALSE and fix.marks = FALSE. The cross-type pair-correlation function $g_{AB}(r)$ was calculated using pcfcross, with Ripley’s isotropic edge correction, an Epanechnikov kernel, stoyan = 0.15, bw = NULL and divisor = “r”. The first input pattern was marked A and the second B; pcfcross used A as the source class and B as the target class. For each observed or simulated pattern, the default bandwidth rule was applied to its target-class intensity: $h=\frac{0.15}{\sqrt{{\hat{\lambda }}_{B}}}$, with $bw=\frac{h}{\sqrt{5}}$. Thus, the PCF bandwidth was recalculated using the simulated B count in each replicate, while the intensities used to generate the Poisson simulations remained fixed.

Observed and simulated functions were evaluated using the same distance grid, with 0.05 m increments in the supplied code. Figure 8 displays site-specific ranges of 0–18 m for a1, 0–13 m for a2, and 0–25 m for a3 and a4. At each distance, the lower and upper envelopes were the minimum and maximum of the 99 simulated values (nrank = 1, global = FALSE, VARIANCE = FALSE). The theoretical cross-type reference was $g_{AB}(r)$ = 1. These are pointwise simulation envelopes, not 95% confidence bands or a global test over the entire distance interval. Departures were interpreted as deviations from the specified homogeneous Poisson reference; they cannot by themselves establish facilitation, competition, or environmental causes. Calculations used R and spatstat.

## 5. Conclusions

This study described crown-area size classes and spatial point patterns of *K. ceratoides* at four sites, without independent age validation or evidence of population trajectories. Some plots showed clustering at short distances and often fell within simulation envelopes at larger distances; this does not establish stress resistance or an elevation effect. Several point patterns fell within inhomogeneous Poisson and Thomas process envelopes, but model fit does not identify habitat heterogeneity or dispersal limitation as causes. Spatial point patterns differed among small, intermediate, and large classes over the distances reported; the mechanisms responsible remain to be tested. Some size–class pairs showed positive deviations at short distances and negative deviations at longer distances within individual plots; these deviations do not establish facilitation, competition, or adaptation. The findings offer a descriptive baseline for future replicated studies of alpine cushion-plant size and spatial structure. Future conservation monitoring in the Tibetan antelope breeding grounds could be informed by replicated site surveys, measured environmental covariates, independent size–age validation, and fully documented point-pattern fitting procedures.

## Figures and Tables

**Table 1:** Population characteristics of each sample plot.

Sampling Areas	Average Crown Diameter/cm	Plant Height/cm	Average Surface Area/cm^2^
S1	14.95	3.11	187.52
S2	16.4	2.77	220.97
S3	16.19	2.83	220.71
S4	15.19	1.17	194.79

**Table 2:** Geographic location of sample plots.

Sampling Areas	Longitudes of Site Center (E)	Latitudes of Site Center (N)	Elevations/m
S1	83°19′19.596″	36°25′3.475″	4184
S2	82°24′25.654″	35°58′9.746″	4548
S3	82°21′49.165″	35°56′19.036″	4855
S4	83°0′37.447″	35°56′58.085″	5124

**Table 3:** Gaussian intensity bandwidths used in the recomputed size–class HP analyses; values are in projected metres.

Site	Size Class	Observed n	Gaussian *σ* (m)
a1	Small	50	2.170730
a1	Intermediate	108	2.168340
a1	Large	13	1.962006
a2	Small	36	1.461071
a2	Intermediate	210	1.586303
a2	Large	42	1.523463
a3	Small	95	3.061286
a3	Intermediate	333	3.061286
a3	Large	69	3.062840
a4	Small	126	3.005626
a4	Intermediate	315	3.097489
a4	Large	36	2.857905

## Data Availability

Data are contained within the article.

## Abbreviations

The following abbreviations are used in this manuscript:

CSR	Complete Spatial Randomness
HP	Inhomogeneous Poisson Process
HT	Homogeneous Thomas Process
DOM	Digital Orthophoto Maps
DSM	Digital Surface Models
UAV	Unmanned Aerial Vehicle
